# Facile Assessment of Protein Binding Events Using a Colorimetric Dye

**DOI:** 10.3390/s26206643

**Published:** 2026-10-21

**Authors:** Ayza Croskey, Christopher A. Azaldegui, Clint J. Regan, Anna K. Mapp, Raychelle Burks

**Affiliations:** 1 Program in Chemical Biology, University of Michigan, Ann Arbor, MI 48109, USA; cayza@umich.edu (A.C.); azaldeg@stanford.edu (C.A.A.); 2 Life Sciences Institute, University of Michigan, Ann Arbor, MI 48109, USA; 3 Department of Chemistry, American University, Washington, DC 20016, USA

**Keywords:** low-cost screening, dynamic proteins, colorimetric assay

## Abstract

**Highlights:**

**What are the main findings?**

- The inexpensive colorimetric dye erythrosin B responds to dynamic protein concentration and ligand binding events.
- The colorimetric responses with erythrosin B are comparable to those observed in fluorescence assays such as differential scanning fluorimetry.

**What are the implications of the main findings?**

- With a low price point and minimal instrumentation requirements, the erythrosin B colorimetric assay provides an accessible and sustainable entry into studies of dynamic proteins.
- The results with erythrosin B suggest that other colorimetric dyes could be deployed in a similar fashion.

**Abstract:**

Colorimetric techniques and their associated dyes can offer lower-cost and more accessible alternatives than their fluorescent and luminescent counterparts for many screening applications. Here, we sought to identify colorimetric dyes suitable for a presumptive assay probing protein–ligand binding, with a particular focus on conformationally dynamic proteins that are often recalcitrant to standard biophysical methods. From an array of 23 commercially available colorimetric dyes, we identified three candidates that exhibited concentration-dependent absorbance changes in the presence of two structurally distinct proteins of interest, Med25 AcID and CBP KIX. Follow-up studies of protein–ligand binding events narrowed the focus to erythrosin B, which was the most sensitive reporter of Med25 and CBP binding events with existing small-molecule ligands. Notably, binding data for Med25–norstictic acid and CBP–garcinolic acid complexes exhibited clustering patterns consistent with orthogonally measured thermostability data for each complex. Thus, colorimetric dyes such as erythrosin B may serve as more broadly accessible tools for presumptive screening assays of high-value protein targets, including those with a high degree of conformational malleability.

## 1. Introduction

More than 50% of the eukaryotic proteome contains intrinsically disordered regions (IDRs) and is conformationally dynamic. In cellular processes, from transcription to protein folding, conformational mobility is intrinsic to function, and the prevalence of such proteins in pathological states motivates their study and targeting [1,2,3,4,5,6]. For example, unregulated formation of the IDR-rich and conformationally dynamic ETV5-Med25 complex in BRAFV600E-driven melanomas underpins aggressive disease, making it an attractive therapeutic target, yet progress towards this goal has been slow [7,8,9,10,11]. Dynamic proteins such as ETV5 and Med25 are often especially challenging to study and target because many commonly used biophysical and biochemical assays were developed for proteins with stable three-dimensional structures [12,13]. We were thus motivated to identify accessible and suitably sensitive alternatives for detecting the binding events of conformationally dynamic proteins of interest.

**Figure 1:**
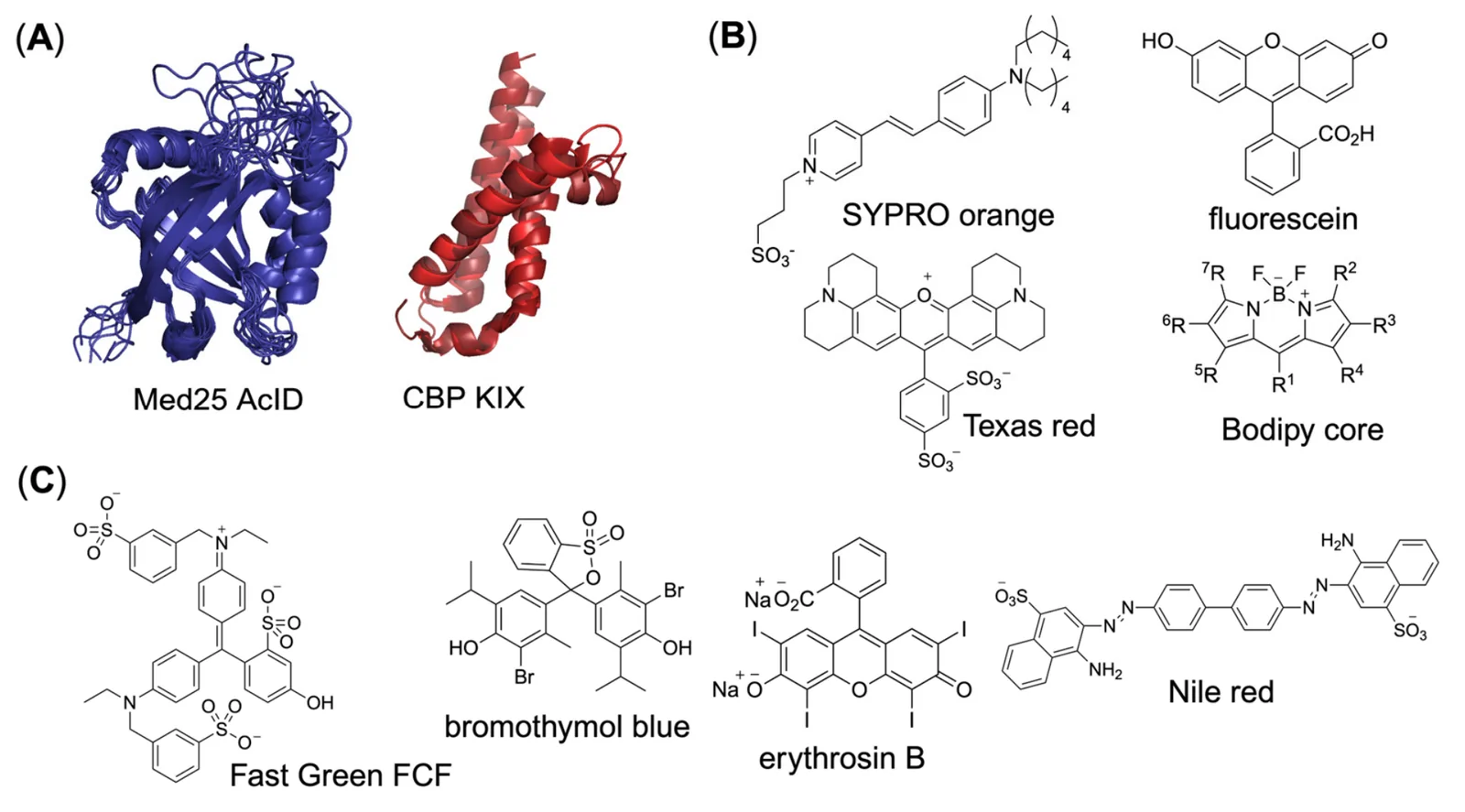
(**A**) Representative dynamic proteins Med25 AcID: PDB ID 2XNF and CBP KIX: PDB 4I9O (dark red), PDB 6DNQ (med red), and PDB 2LQH (light red). The Med25 AcID structure is the superimposition of 10 distinct poses from NMR structure 2XNF, revealing the high mobility of specific substructures [36]. The CPB KIX depiction is the superimposition of KIX bound to a small-molecule ligand (dark red) [33], the viral transcriptional protein HBZ (medium red) [37], or the transcription factor FOXO3a (light red) [38]. Notably, the loop connecting two helices has a distinct structure in each of the bound forms of KIX. (**B**) Representative fluorescent dyes used to screen and study biomolecules. (**C**) Colorimetric dyes used in a variety of sensing applications [18].

Fluorometric dyes are valuable reagents in assays of canonical and dynamic proteins (Figure 1B). In differential scanning fluorimetry (DSF), for example, a change in signal intensity of the environmentally sensitive fluorometric dye SYPRO orange upon thermally induced protein unfolding enables rapid measurement of protein stability in the presence and absence of a binding partner [14,15,16,17]. We noted the structural similarities of SYPRO orange and related fluorometric dyes with colorimetric dyes (Figure 1C). These colorimetric dyes are highly effective agents in low-cost, rapid, and sensitive assays for applications including clinical diagnostics, environmental monitoring, food safety, research diagnostics, and forensic screening [18,19,20,21,22,23,24,25,26,27]. In many cases, engagement with biomolecules—including proteins—is intrinsic to the use of colorimetric dyes in such applications [18]. We hypothesized that a colorimetric assay featuring such dyes could provide a more accessible strategy for preliminary screening of conformationally dynamic proteins with potential binding partners. Screening assays occupy a vital place in an analytical workflow, enabling users to appropriately leverage their limited resources on samples assessed to need additional examination [28].

To test our hypothesis, we chose two protein motifs representative of the challenges and opportunities of dynamic proteins as our targets, Med25 AcID and CBP KIX (Figure 1A) [9,10,29,30,31]. Although structurally distinct, Med25 AcID and CBP KIX engage disordered transcription factor motifs as part of transcriptional initiation and are frequently dysregulated in disease. Both proteins exhibit considerable conformational plasticity and interact with transcription factors and smaller molecules with modest affinities [29,30,32,33,34]. Thus, many common presumptive assays of protein binding interactions are ineffective or difficult to adapt to Med25 AcID and CBP KIX. Here, we report that erythrosin B, a low-cost, commercially available colorimetric dye, can be used as an accurate reporter of specific binding interactions in a presumptive assay with these two dynamic proteins. The resulting absorbance assay is inexpensive and microplate reader-compatible, and it provides a practical strategy to rapidly indicate interactions of dynamic proteins without requiring specialized fluorescence instrumentation [9,35].

## 2. Materials and Methods

### 2.1. Assay Materials

Colorimetric dyes were used as received from commercial sources as detailed in the Supplemental Information (SI) (SI Figures S1–S3 and SI experimental procedures). Inhibitors garcinolic acid (MicroSource Discovery Systems, Inc., Gaylordsville, CT, USA) and norstictic acid (Cayman Chemical, Ann Arbor, MI, USA) were purchased and stored as recommended. The Med25 expression plasmid [pAcID(394-543)-His6] was generously provided by Prof. Patrick Cramer and expressed as previously described [36]. CBP KIX (586-672) was expressed and purified from heat-shock-competent BL21-AI *E. coli* in Terrific Broth (TB) containing ampicillin and tetracycline, as previously described [35].

### 2.2. Colorimetric Assay Development and Data Analysis

For absorbance assays of protein and dye, protein (Med25 AcID or CBP KIX) at a concentration of 32 μM was incubated with 10 μM dye in Med25 buffer (10 mM sodium phosphate buffer pH 6.8, 100 mM NaCl, 0.001% NP-40, 10% glycerol, 1 mM DTT) or CBP KIX buffer (25 mM sodium phosphate buffer pH 7.8, 125 mM NaCl, 0.001% Triton X-100, 1 mM DTT) in a clear, low-volume 384-well plate (Greiner Bio-One, Monroe, NC, USA). Protein-dye samples (final volume 20 μL) were incubated at room temperature (RT) for 30 min. Following incubation, the plate was centrifuged at 500× *g* for 1 min and absorbance was then read from 390 to 750 nm every 5 nm using a BioTek Synergy H1 multimode reader (Agilent Technologies, Santa Clara, CA, USA). All absorbance spectra were plotted using GraphPad Prism 10 (GraphPad Software, Boston, MA, USA).

For absorbance assays of protein, inhibitor, and dye, a 10 mM solution (DMSO) of either garcinolic acid or norstictic acid was diluted in the relevant protein buffer such that the final concentration of DMSO in each assay well would be 5%. The order of addition to each well was protein (final concentration 32 μM), inhibitor, and erythrosin B (final concentration 10 μM). The resulting solutions (final volume 20 μM) were incubated at RT for 1 h. Following incubation, the plate was centrifuged at 500× *g* for 1 min and absorbance was then read from 390 to 750 nm every 5 nm using a BioTek Synergy H1 multimode reader. All absorbance spectra were plotted using Graphpad Prism 10.

Principal Component Analysis (PCA) was performed using Python 3.12 (Python Software Foundation, Beaverton, OR, USA) as described in the Supporting Information. To evaluate the suitability of the selected principal components, the explained variance ratio and cumulative explained variance were calculated for all principal components. Technical reproducibility was assessed by calculating the coefficient of variation (CV%) among replicate measurements within each experimental group. Cluster quality in the PCA score space was assessed using the silhouette score. Multivariate Analysis of Variance (MANOVA) was performed using the first two principal component scores as dependent variables and sample group as the categorical factor. Reproducibility assessment, silhouette score determinations, and MANOVA were all done using standard R packages (R Foundation, Vienna, Austria) and the results of the assessment are shown in each relevant figure.

### 2.3. Thermostability Assay Application

Thermostability assays were conducted in triplicate utilizing PCR strip tubes sealed with clear cap strips. In total, 8 μM Med25 AcID or 15 μM CBP KIX was incubated with norstictic acid or garcinolic acid at the indicated concentrations at RT for 30 min, and then 5× SYPRO orange dye (1:100 dilution of purchased 5000× stock; Invitrogen, Thermo Fisher Scientific, Waltham, MA, USA) was added to the mixture. DSF analysis was performed using an Applied Biosystems StepOnePlus qPCR (Thermo Fisher Scientific, Waltham, MA, USA) to obtain melting curves by exciting at 488 nm and monitoring emission at 602 nm over a temperature gradient of 25–95 °C with a 1 °C/min ramp. Raw fluorescence data (RFU) was imported into the online data analysis program DSFworld and Tm was calculated using a sigmoidal fitting model as previously described [39]. All melting temperature changes were plotted using Graphpad Prism 10.

## 3. Results and Discussion

**Figure 2:**
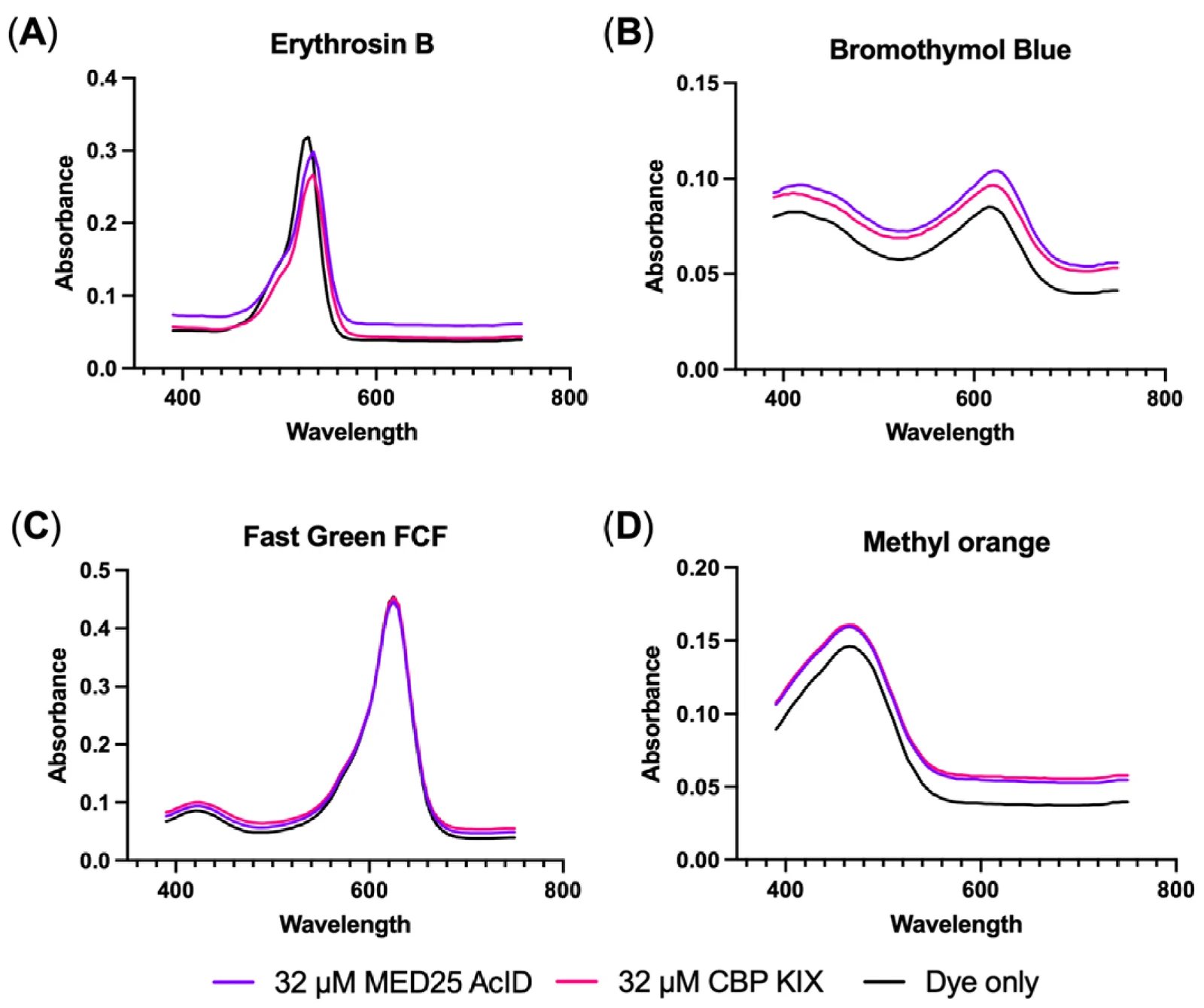
Absorbance spectra for the three dyes with the negative control dye display the extent of target differentiation. The spectra for (**A**) erythrosin B, (**B**) bromothymol blue, (**C**) fast green FCF, and (**D**) methyl orange were measured from 350 to 750 nm and plotted in GraphPad Prism. Each curve represents the average of five independent replicates.

To identify candidate dyes for the planned colorimetric binding assay, we first measured the absorbance spectra of an array of twenty-three structurally diverse and commercially available dyes in the presence of increasing concentrations of CBP KIX and Med25 AcID (Figure 1A, Figures S1 and S2). From that experiment, erythrosin B, bromothymol blue, and fast green FCF emerged as candidates for further analysis as they exhibited reproducible protein concentration-dependent spectral changes that were distinct for CBP KIX and Med25 AcID (Figure 2 and Figure S3). The presence of CBP KIX or Med25 AcID produces a 5 nm shift in the λ_max_ accompanied by a small decrease in peak intensity for the xanthene dye erythrosin B (Figure 2A). These changes are consistent with the bathochromic and hypochromic shifts observed in previous studies of erythrosin B–protein binding [40,41,42,43]. This was not the case for the other xanthene dye present in the initial array, fluorescein. The lack of a significant colorimetric response with fluorescein is not unexpected, as interaction with proteins such as bovine serum albumin is typically detected through fluorescence measurements [44].

The two other candidate dyes produce distinct spectral changes in the presence of CBP KIX and Med25 AcID. Bromothymol blue exhibits hypochromic shifts in the entire spectrum when either protein is present and a small bathochromic shift with Med25 AcID (Figure 2B). Bromothymol blue was the sole sulfonephthalein dye out of five in the array exhibiting a significant spectral change (Figure S2), consistent with prior studies of this dye family [45]. Changing the buffer pH would likely increase the spectral response accompanying protein binding, but CBP KIX and Med25 AcID are, like many dynamic proteins, sensitive to pH changes [45,46,47]. The triarylmethane dye fast green FCF displays small shifts on the perimeter of the absorbance peak when combined with CBP KIX and Med25 AcID (Figure 2C) [48]. As with bromothymol blue, the spectral response with protein binding is highly pH-dependent. These results were compared with those of methyl orange, a dye that showed similarly minimal spectral changes with CBP KIX and Med25 AcID in the initial array. As shown in Figure 2D, in follow-up experiments, methyl orange displayed little differentiation between Med25 AcID and CBP KIX. To explore target differentiation among the dyes, PCA was used to reduce the spectral data and reveal possible associations (Figure S3). Erythrosin B emerged as the dye that produces discrete clusters for each condition of CBP KIX and Med25 AcID, resulting in the selection of this dye as the candidate for additional investigation.

**Figure 3:**
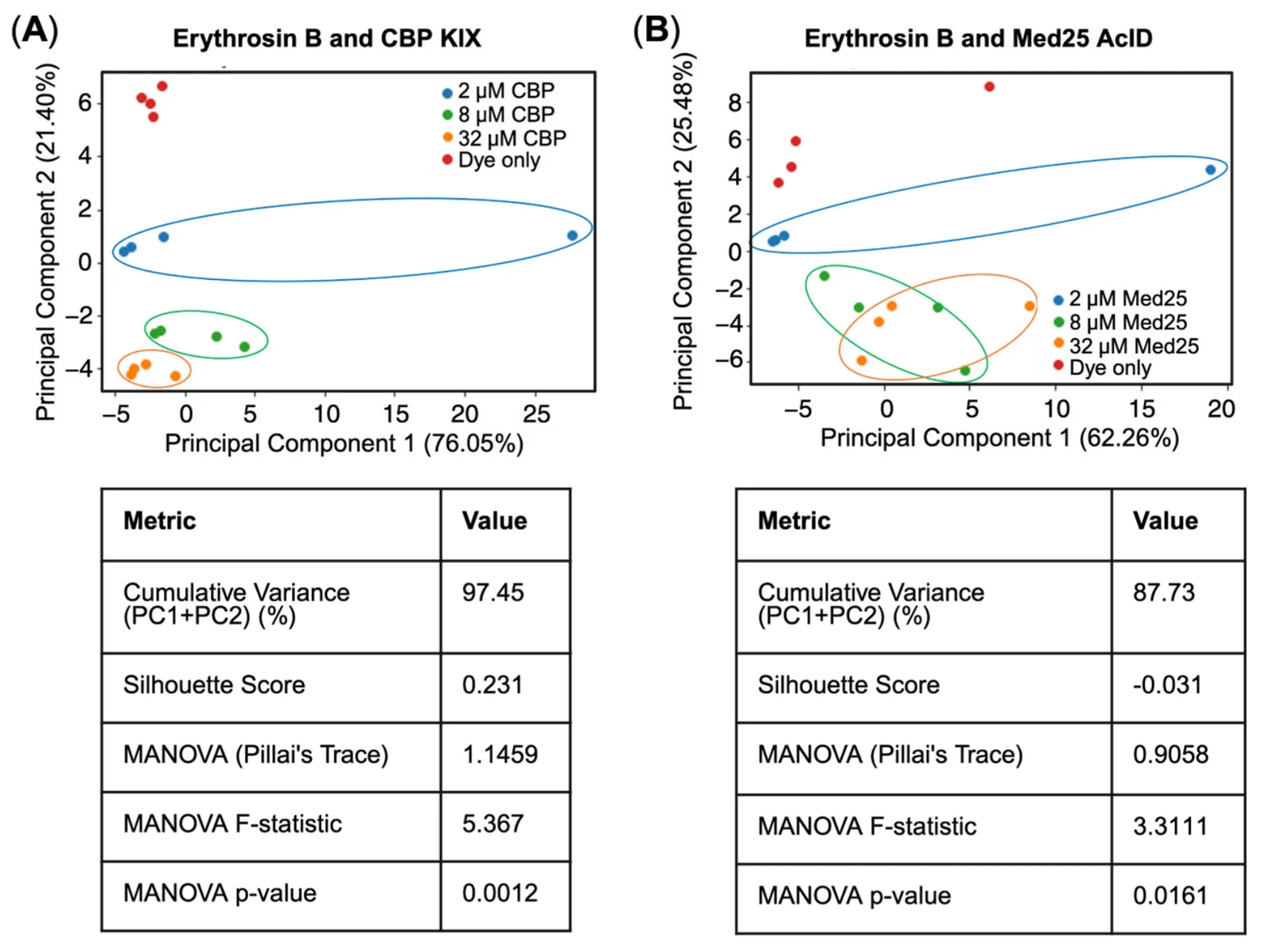
Erythrosin B absorbance changes with protein concentration. (**A**) PCA plots of erythrosin B absorbance data with supporting validation metrics in the presence of CBP KIX, illustrating distinct clusters at each concentration of CBP KIX. (**B**) PCA plots of erythrosin B absorbance data with supporting validation metrics in the presence of Med25 AcID, indicating Med25 AcID concentration dependence of erythrosin B absorbance. Each point on the PCA plot represents a single well and each condition was tested in quadruplicate.

Next, we assessed erythrosin B spectral changes over a range of protein concentrations to determine conditions that maximize the spectral response. To this end, spectral changes with protein concentrations up to 32 μM were tested in quadruplicate with 10 μM erythrosin B. No evidence of protein precipitation or aggregation was observed over the entire concentration range. Spectral data were evaluated by PCA, showing that there is a protein concentration-dependent spectral response for both Med25AcID and CBP KIX. For CBP KIX, erythrosin B produces distinct clusters for each concentration (Figure 3A). Although the silhouette score (0.231) indicates incomplete separation of individual observations in the PCA space, the MANOVA results indicate that the sample groups possess different and significant multivariate spectral characteristics. Similar to the CBP KIX data (Figure 3B), the spectral responses at all concentrations of Med25 AcID were distinct relative to dye-only responses. However, the Med25 AcID data at 8 μM and 32 μM overlap, suggesting signal saturation at the higher protein concentrations. This is reflected in the low silhouette score (Figure 3B) and, as with the CBP KIX data, the results of the MANOVA analysis indicate that the sample groups display significantly different multivariate spectral characteristics.

**Figure 4:**
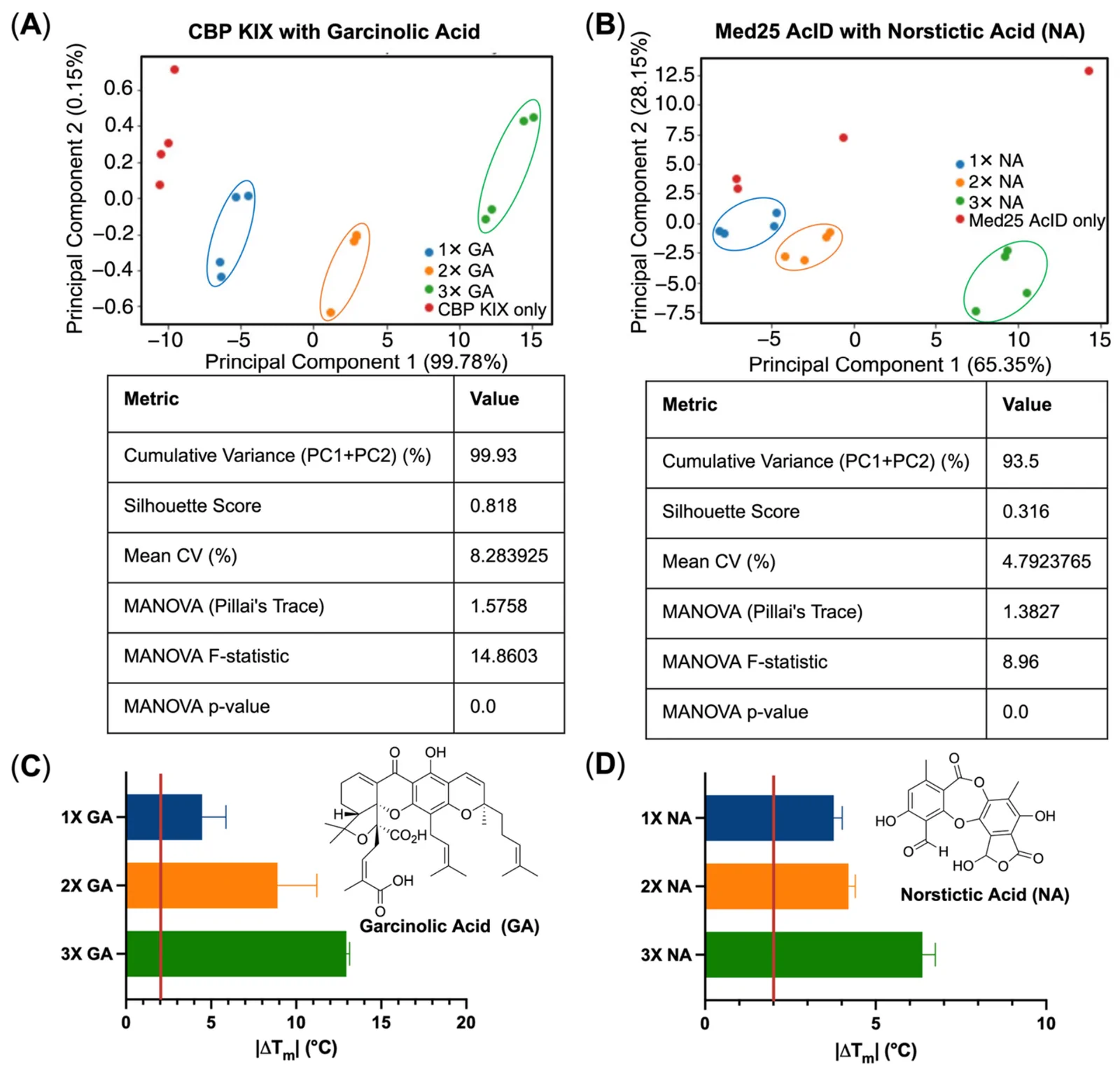
The erythrosin B colorimetric assay detects small-molecule binding to CBP KIX and Med25 AcID. (**A**,**B**) The natural products garcinolic acid (GA) and norstictic acid (NA) are well-characterized ligands for CBP KIX and Med25 AcID, respectively. Formation of CBP KIX•GA and Med25 AcID•NA complexes was detected in the erythrosin B colorimetric assay using the conditions of Figure 3. In this format, protein (32 μM) and erythrosin B (10 μM) were held constant, while equivalents of the small-molecule ligand were increased. The clustering patterns for the erythrosin B absorbance data with CBP KIX•GA: and Med25 AcID:NA reveal a dose-dependent response. Each point on the PCA plots represents a single well; thus, each condition was tested in quadruplicate. (**C**,**D**) Using previously established protocols, formation of CBP KIX•GA and Med25 AcID•NA complexes was detected using differential scanning fluorimetry with SYPRO orange as the dye. In this assay, protein concentration (CBP KIX: 8 μM; Med25 AcID: 15 μM) and dye (5× protein concentration) were held constant while ligand concentration was varied as indicated. The red line indicates the threshold of significance and change in temperature was measured in degrees Celsius. The standard deviation for the absolute change in melting temperature (|DTm|) is indicated by the error bars resulting from three independent replicates.

Attention then turned to testing erythrosin B in a presumptive binding assay format with both protein targets. As noted earlier, there are well-characterized small-molecule ligands for Med25 AcID and CBP KIX suitable for benchmarking this colorimetric assay against established strategies. Garcinolic acid (Figure 4) engages CBP KIX with a K_D_ of 1 μM and, through a mixed allosteric and orthosteric mechanism, inhibits KIX–transcription factor interactions in vitro and in cells [35]. Norstictic acid (Figure 4) is a covalent ligand of Med25 AcID with an IC_50_ of 4 μM, selectively forming a Schiff base with a dynamic, lysine-rich loop. Norstictic acid inhibits binding events at both binding surfaces in Med25 in vitro and in cells [9]. Both garcinolic acid and norstictic acid produce conformational changes in their respective protein binding partners, and thus, we hypothesized that erythrosin B would exhibit a spectroscopic change for ligand-bound Med25 AcID or CBP KIX relative to unbound protein.

To test the utility of the colorimetric assay for detecting ligand–protein binding events, we directly compared colorimetric results to those obtained with differential scanning fluorimetry (DSF). As shown in Figure 4A, the assay conditions of Figure 3 (10 μM erythrosin B, 32 μM CBP KIX) were used in binding assessments of garcinolic acid, with the resulting absorbance spectra evaluated by PCA. With a silhouette score of 0.818 and a highly significant effect of sample identity on the PCA scores as indicated by MANOVA, the addition of garcinolic acid resulted in high within-group similarity and strong between-group separation. The same assay conditions were then applied to Med25 AcID and norstictic acid, and dose-dependent changes were observed (Figure 4B). There was less separation between clusters relative to CBP KIX, as indicated by the silhouette score of 0.316. However, as in the data of Figure 3, the MANOVA again confirmed a statistically significant effect of sample identity on the multivariate PCA scores. And, in both the Med25 AcID and CBP KIX experiments, distinct changes were observed at a single equivalent of ligand, with clear dose dependence.

In parallel, DSF was carried out with CBP KIX•garcinolic acid and Med25 AcID•norstictic acid pairings under previously reported conditions [9,35]. As shown in Figure 4C,D, norstictic acid and garcinolic acid modify the thermostability of their respective targets in a dose-dependent manner, as detected by changes in SYPRO orange fluorescence. Considered together, the erythrosin B colorimetric assay provides results analogous to those of the more established DSF assay.

Taken together, the data reported here indicate that the changes in erythrosin B absorbance in the presence of different proteins (CBP KIX, Med25 AcID), altered protein concentrations, and the formation of Med25 AcID•norstictic acid and CBP KIX•garcinolic acid complexes result from engagement with the proteins. This is consistent with erythrosin B’s widespread use as a dye in cellular and biochemical experiments [49,50,51]. Erythrosin B has been noted for its ‘promiscuous protein binding ability’, which makes it suitable to serve as a broad-range reporter in colorimetric protein binding assays [40,41]. Like the popular DSF reporter SYPRO orange, it binds hydrophobic protein regions and is sensitive to the polarity of its environment [16,40,52].

The erythrosin B colorimetric assay is a presumptive test, suitable for providing an initial signal of protein–ligand binding interactions worthy of additional investigation. The capabilities shown here can support accessibility in biochemical screening. Cost-effective and fit-for-purpose screening practices have been highlighted as integral to sustainability in fields like forensics [21], in which colorimetric dyes are commonly used. Erythrosin B costs ~$150 for 100 g and, compared to the ~$300 for 500 mL cost of the popular DSF dye SYPRO orange, the erythrosin B assay detailed herein mirrors the stabilizing shifts seen in SYPRO orange-mediated DSF. The instrumentation needed for UV assays carried out at ambient temperature is typically less costly compared to that needed for fluorescence-based assays such as differential scanning fluorimetry that require variable temperature control. The Bryonoy UV-vis 96-well plate reader, for example, has a small footprint (9.6 × 15.4 × 5.5 cm), is portable, and comes in at just under $5000 including software (Quote #AN26020407 received 3 February 2026). Differential scanning fluorimetry is often carried out using benchtop real-time quantitative PCR instruments which range in cost from $10,000 to $30,000. Thus, the use of colorimetric dyes for protein characterization and/or screening approaches provides an accessible alternative to techniques such as DSF for entry-level, presumptive screening within an analytical workflow. Rather than being a replacement for fluorescence assays such as DSF, we posit that a colorimetric assay like the one presented here lowers the ramp of entry to a robust analytical workflow. A lower-cost screening starting point can provide critical information to aid analysts in determining which ligands to advance through their analytical workflows and which to divert, likely conserving resources.

## 4. Conclusions

Using data derived from absorbance spectra, readily available colorimetric dyes were assessed as ligand binding event reporters for the proteins Med25 AcID and CBP KIX. Of the three identified dyes, erythrosin B displayed versatility against the protein targets and the capability to detect ligand-induced stabilization. In addition, this colorimetric dye proved to be a lower-cost analytical workflow entry point than a more established DSF technique. There are limitations to the erythrosin B-based assay format described here. Thus far, it has been demonstrated on two different dynamic proteins only, and the extent to which it can be used for the broader class remains to be defined. In addition, the assay is formatted for assessment of the isolated proteins of high purity to minimize confounding background interactions and in its current form is not unlikely to be applicable to complex lysates or similar heterogeneous milieu. Moving forward, a broader screening campaign to identify colorimetric dyes with even more robust spectral responses, alone or as an array [24,53], will expand the utility of the approach beyond presumptive applications.

## Data Availability

The raw data supporting the conclusions of this article will be made available by the authors on request.

## Supplementary Materials

The following supporting information can be downloaded at: https://www.mdpi.com/article/10.3390/s26206643/s1.

## Abbreviations

The following abbreviations are used in this manuscript:

KIX	Kinase-Inducible Domain
AcID	Activator Interaction Domain
CBP	CREB-Binding Protein
Med25	Protein #25 in the coactivator complex named Mediator
DSF	Differential Scanning Fluorimetry
IDR	Intrinsically Disordered Region
NMR	Nuclear magnetic resonance
HTS	High-throughput screening
FP	Fluorescence polarization
