# Supplementary material for: Facile Assessment of Protein Binding Events Using a Colorimetric Dye
Source: Sensors (Basel). 2026 Oct 21;26(20):6643. doi: 10.3390/s26206643 (PMC13709039; doi:10.3390/s26206643)
Supplement: Supplementary file 1 [file sensors-26-06643-s001.zip › sensors-4505409-supplementary.pdf]

Supplemental Information

Facile Assessment of Protein Binding Events Using a Colorimetric Dye

Ayza Croskey [1], Christopher A. Azaldegui [1], Clint J. Regan [2], Anna K. Mapp [1,2,*] and Raychelle Burks [3,*]

[1] Program in Chemical Biology, University of Michigan, Ann Arbor, MI 48109, USA
[2] Life Sciences Institute, University of Michigan, Ann Arbor, MI 48109, USA
[3] Department of Chemistry, American University, Washington, DC 20016, USA
* Correspondence: amapp@umich.edu (A.K.M.); burks@american.edu (R.B.)

Contents of Supplemental Information:

	Page
**Experimental procedure for colorimetric dye screening, data analysis**	S2
**Figure S1:** The twenty-three structurally diverse dyes utilized in the colorimetric array screening	S3
**Figure S2:** Dye concentrations in the colorimetric screening assay	S3
**Figure S3:** Identification of dye 'hits' from the colorimetric screen	S4
**Figure S4:** PCA of absorbance spectra for Med25 AcID and CBP KIX in complex with four dye hits	S5

Screens of the colorimetric dye array and data analysis

Colorimetric array experiments were carried out in clear, low-volume 384 well plate (Greiner Bio-One 784101) to which protein (Med25 AcID or CBP KIX) in either Med25 buffer (10 mM sodium phosphate buffer pH 6.8, 100 mM NaCl, 0.001 % NP-40, 10% glycerol,1 mM DTT) or CBP KIX buffer (25 mM sodium phosphate buffer pH 7.8, 125 mM NaCl, 0.001 % Triton X-100, 1 mM DTT) were combined with each of the 23 dyes dissolved in PBS pH 7.4 with .5% DMSO with a final volume per well of 20 mL. The final protein concentration in each well was 1.1 mM, 2.2 mM, 4.4 mM, 8.75 mM, or 17.5 mM, such that each dye was assessed over a 5-point concentration range in triplicate. The final concentration of each dye is shown in Figure S2; the concentrations were chosen such that the maximum absorbance for each dye was 0.5. In addition, buffer-only wells were used as negative controls. The 384-well plate was kept in the dark at room temperature for 30 min before collecting absorbance spectra. Absorbance spectra from 390-700 nm at 5 nm intervals were collected using a BioTek Synergy H1 multimode reader.

Data analysis

RGB analysis was completed utilizing Python to convert the spectral data from each well into a corresponding RGB value. The no-protein control wells for each dye-protein pair were set as the initial RGB value. The value of RGB_Final – RGB_Initial for each well was then calculated to create a difference map, as shown in Figure S3A. From this analysis, dye #8 bromothymol blue and dye #9 fast green FCF were identified as candidates for further investigation.

A second analysis examined the change in dye absorbance as specific wavelengths as protein concentration changed (Figure S3B). The triplicate protein-dye absorbance data at each recorded wavelength was averaged and the absorbance for the corresponding control was subtracted. For each wavelength, a simple linear regression was carried out with the background-subtracted absorbance values of the various concentrations of protein. The change in wavelength absorbance was then defined as the maximum linear slope. Dye #17, erythrosin B, demonstrated a maximum absorbance change at 550 nm that was two standard deviations above the mean for the maximum changes of all 23 dyes. With a concentration-dependent change in absorbance that is statistically significant, erythrosin B was also chosen as a candidate dye.

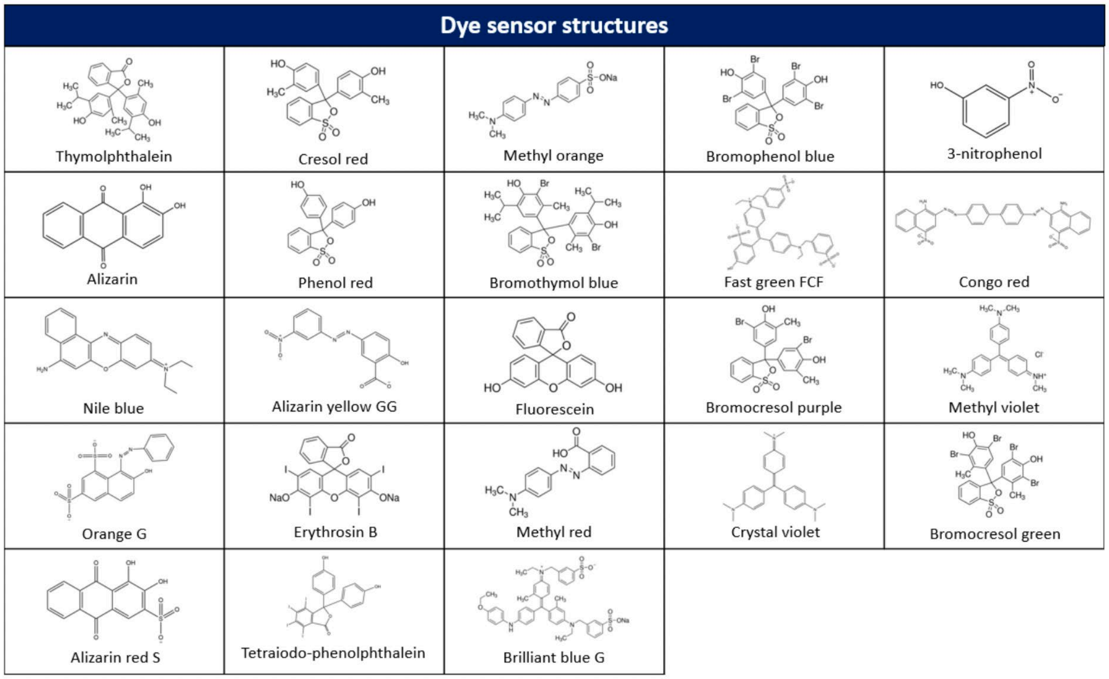

Figure S1 Structures of the dyes utilized in the colorimetric array screening

Dye number	Dye name	Final [uM]
1	Thymolphthalein	12.65
2	Cresol Red	10.66
3	Methyl Orange	10.25
4	Bromophenol Blue	6.42
5	3-Nitrophenol	485.71
6	Alizarin	33.03
7	Phenol Red	8.20
8	Bromothymol Blue	27.27
9	Fast Green FCF	4.45
10	Congo Red	13.14
11	Nile Blue	2.37
12	Alizarin yellow GG	39.62
13	Fluorescein	6.35
14	Bromocresol Purple	22.62
15	Methyl Violet	7.90
16	Orange G	35.09
17	Erythrosin B	5.48
18	Methyl Red	24.75
19	Crystal Violet	5.66
20	Bromocresol Green	26.47
21	Alizarin Red S	41.23
22	Tetraiodo-phenolphthalein	49.07
23	Brilliant Blue G	11.15

Figure S2: Final concentrations of each dye in the colorimetric array. The concentration of each dye was chosen such that the baseline absorbance value would be approximately 0.5 to provide a suitable dynamic range for array analysis.

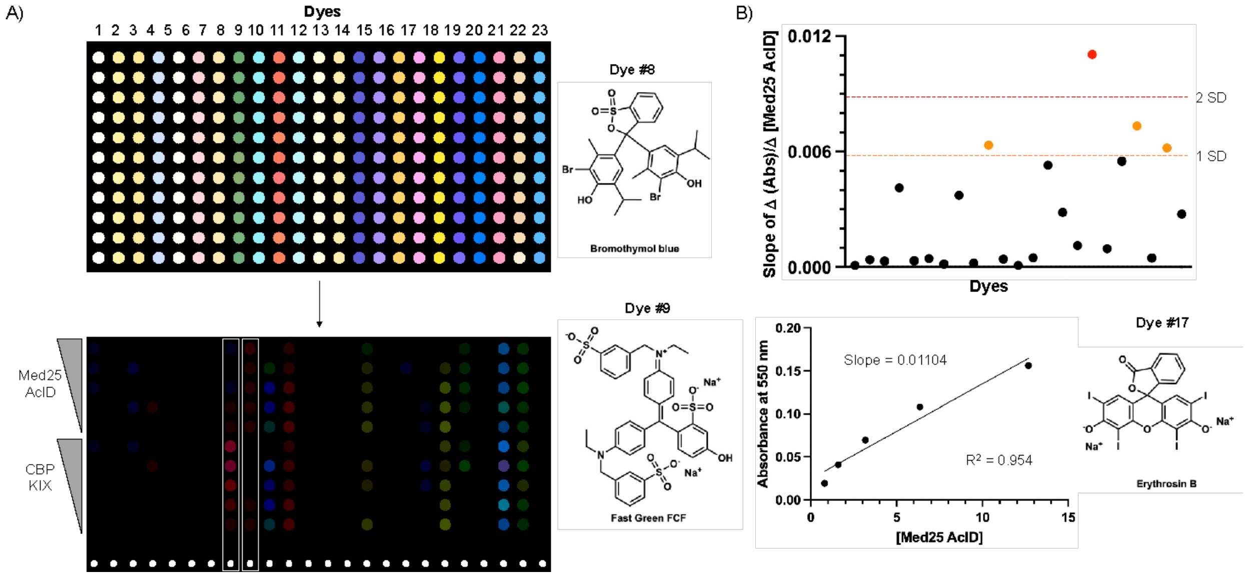

95% confidence interval
Slope 0.006577 to 0.01551
Y-intercept -0.004702 to 0.05376
X-intercept -7.561 to 0.3278

Figure S3 Analysis of colorimetric dye array screening data lead to three candidate dyes for further evaluation. A) RGB analysis performed on the absorbance data from colorimetric arrays treated with either DMSO, CBP KIX, or Med25 AcID identified two dyes that interacted with either CBP KIX (#8, bromothymol blue) or Med25 AcID (#9, Fast Green FCF. B) Single point analysis of absorbance changes across Med25 AcID concentrations through a simple linear regression revealed erythrosin B as having more than a two standard deviation (SD) change relative to the average of all 23 dyes.

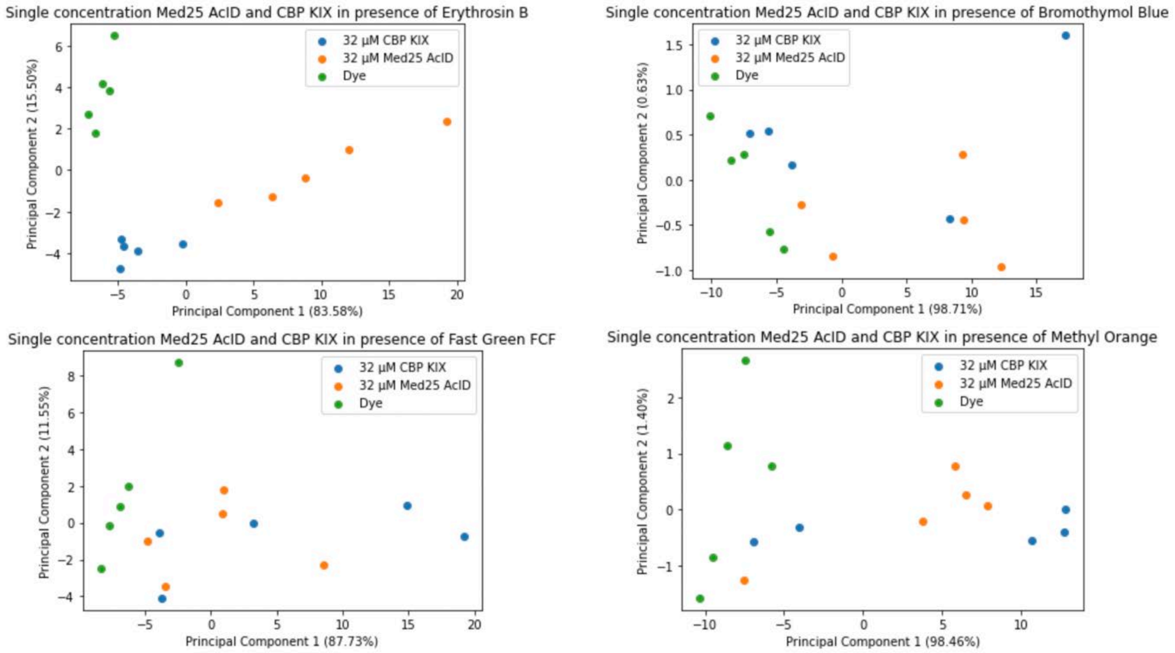

Figure S4 PC Principal Component Analysis (PCA) was performed using Python 3.12 on absorbance data collected (five independent replicates) for each of four dyes in complex with either 32 mM CBP KIX or 32 mM Med25 AcID.
